# Virus‐like particles for vaccination against cancer

**DOI:** 10.1002/wnan.1579

**Published:** 2019-08-27

**Authors:** Mona O. Mohsen, Daniel E. Speiser, Alexander Knuth, Martin F. Bachmann

**Affiliations:** ^1^ The Interim Translational Research Institute “iTRI” National Center for Cancer Care & Research (NCCCR) Doha Qatar; ^2^ Department of BioMedical Research, Immunology RIA University of Bern Bern Switzerland; ^3^ Department of Oncology University of Lausanne Lausanne Switzerland; ^4^ Nuffield Department of Medicine, Jenner Institute University of Oxford Oxford UK

**Keywords:** cancer, vaccine, virus like particles

## Abstract

Active immunotherapy of cancer aims to treat the disease by inducing effective cellular and humoral immune responses. Virus‐like particle‐based vaccines have evolved dramatically over the last few decades, greatly reducing morbidity and mortality of several infectious diseases and expectedly preventing cervical cancer caused by human papilloma virus. In contrast to these broad successes of disease prevention, therapeutic cancer vaccines remain to demonstrate clinical benefit. Yet, several preclinical and clinical trials have revealed promising results and are paving the way for medical breakthroughs. This study reviews and discusses the recent preclinical development and clinical trials in this field.

This article is categorized under:

Biology‐Inspired Nanomaterials > Protein and Virus‐Based Structures

Nanotechnology Approaches to Biology > Nanoscale Systems in Biology

## Graphical abstract

Virus‐like particles, a powerful and flexible platform for the development of cancer vaccines.

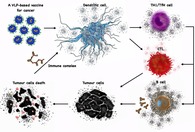

**Abbreviations**
Ab: antibody
ADCC: antibody‐dependent cellular cytotoxicity
APC: antigen‐presenting cell
BCR: B cell receptor
CTL: cytotoxic T‐lymphocyte
CTLA4: cytotoxic T‐lymphocyte‐associated antigen 4
CuMV_TT_: cucumber mosaic VLP incorporating universal tetanus toxoid epitope
DC: dendritic cell
HBcAg: hepatitis B core antigen
HBx: HBV X multifunctional regulatory protein
HCC: hepatocellular carcinoma
HER‐2 or HER‐2/neu: human epidermal growth factor receptor 2
hMSLN: human mesothelin
HPV: human papilloma virus
IBDV: infectious bursal disease virus
LN: lymph node
MHC‐I: major histocompatibility Class I
MHC‐II: major histocompatibility Class II
mMSLN: murine mesothelin
OVA: ovalbumin
PD‐1: programmed cell death protein 1
RHDV: rabbit hemorrhagic disease virus
SHIV: simian‐human immunodeficiency virus
SIV: simian immunodeficiency virus
TACA: tumor‐associated carbohydrates antigen
TLR: toll‐like receptor
Treg: regulatory T cell
TRP2: tyrosinase‐related protein
VLP: virus‐like particle
xCT: cystine‐glutamate exchanging transporter

## 1 INTRODUCTION

### 1.1 Virus‐like particles: A brief summary

**Figure 1:**
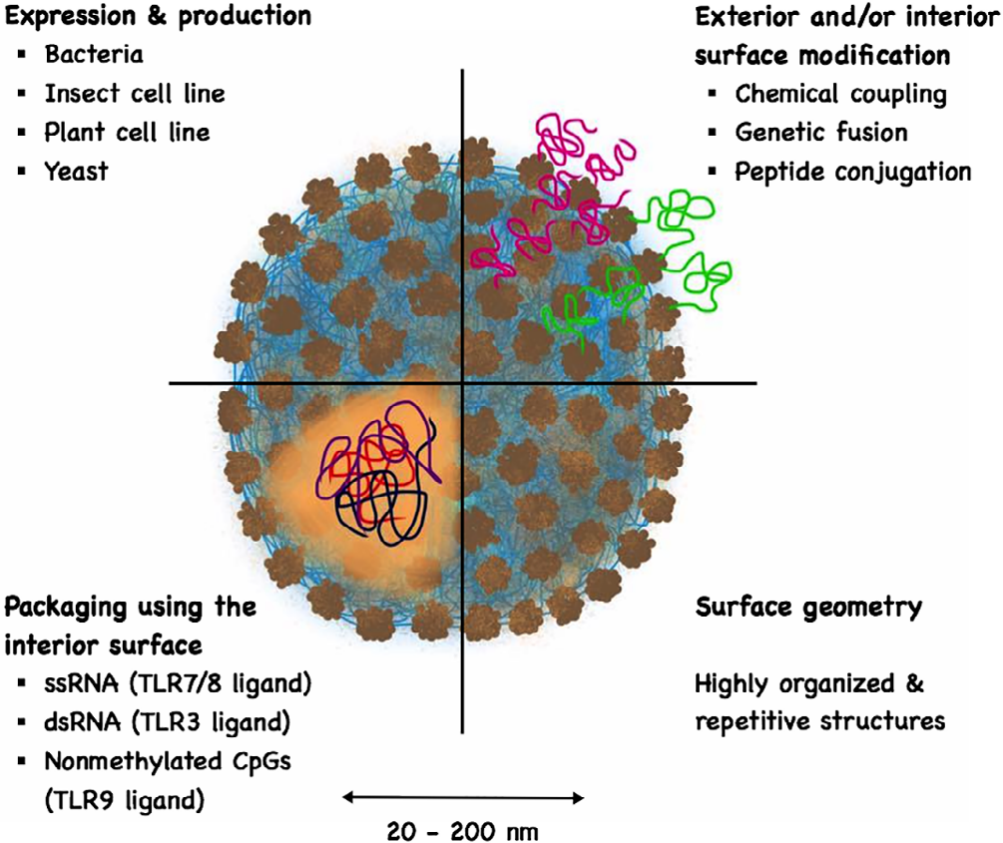
A sketch illustrating some key characteristics of virus‐like particles (VLPs) as an efficient vaccine platform

Virus‐like particles (VLPs) constitute a powerful and flexible platform that harness the immunogenicity of viruses without compromising on safety as VLPs cannot infect nor replicate due to the absence of the viral genome (Chroboczek, Szurgot, & Szolajska, 2014). VLPs may be loaded with innate stimuli, further enhancing their immunogenicity (Storni et al., 2004; Caitlin Lemke, Krieg, & Weiner, 2016; Klimek et al., 2018). Tumor‐specific antigens may be incorporated by genetic fusion or chemical/peptide linkage allowing immunization against peptides, peptide strings or even whole proteins (Mohsen, Zha, Cabral‐Miranda, & Bachmann, 2017). Generally, VLPs have been extensively used as vaccines due to these favorable characteristics. Figure 1 and Table 1 illustrate and summarize some key characteristics of VLPs as an efficient vaccine platform. Further details of VLPs are reviewed elsewhere (Bachmann & Jennings, 2010; Garcea & Gissmann, 2004; Mohsen, Gomes, Vogel, & Bachmann, 2018; Pushko, Pumpens, & Grens, 2013; Schiller & Lowy, 2015).

**Table 1:** Key characteristics of virus‐like particles (VLPs) as an efficient vaccine platform

Characteristics	Description	References
VLPs	Particles that are built‐up and self‐assembled into icosahedral or rod‐shaped structures during the expression of one or several viral‐structural proteins. Mostly, viral capsid or envelope proteins assemble into VLPs but in some cases core proteins also form VLPs. VLPs mimic the structure and symmetry of authentic viruses. They are noninfectious as they lack the proteins and genetic material for replication (replicases and nucleic acids)	Bachmann and Jennings (2010); Pumpens et al. (2009); Roldao, Mellado, Castilho, Carrondo, and Alves (2010); Sander and Lollini (2018)
Size	VLPs range from 20 to 200 nm, a favorable size allowing their free draining into lymph nodes	Cubas et al. (2009); Gomes, Mohsen, and Bachmann (2017) Manolova et al. (2008)
Expression and production	VLPs can be produced in a variety of systems including bacteria, insect or mammalian cell lines, plants or yeast	Arevalo, Wong, and Ross (2016); Santi, Huang, and Mason (2006)
Surface geometry	VLPs have highly organized and repetitive structures that are recognized as potent geometric pathogen‐associated structural patterns (PASP). This does not only lead to efficient cross‐linking of B cell receptors but also recruits members of the innate humoral immune system such as natural antibodies and complements, further enhancing innate and adaptive immune responses	Bachmann and Jennings (2010); Manolova et al. (2008); Rynda‐Apple, Patterson, and Douglas (2014)
Modifying exterior or interior surface	The exterior or interior surface of VLPs can be functionalized and modified to display the antigens or epitopes of interest by different means: Chemical coupling Genetic fusion and engineering Peptide conjugation	Jegerlehner et al., (2002), Brune et al. (2016); Kaczmarczyk, Sitaraman, Young, Hughes, and Chatterjee (2011); Martin Caballero et al. (2012); Pomwised, Intamaso, Teintze, Young, and Pincus (2016); Pumpens (2016); Tegerstedt et al. (2005); Zeltins et al. (2017)
Immunostimulatory molecules	Some VLPs assemble around RNA fragments (noninfectious or replication competent) during the expression process in host cells. VLPs can also be disassembled and reassembled in the presence of different TLR‐ligands such as oligodeoxynucleotides (CpGs) (TLR‐9 ligand), polyGLU, ssRNA (TLR 7/8 ligand) or dsRNA (TLR‐3 ligand)	Dash, Federica, Ottenbrite, and Chiellini (2011); Kawano, Matsui, and Handa (2018); Sioud (2006); Storni et al. (2004); Gomes et al. (2019).

## 2 VLP‐BASED VACCINES AND THE INDUCTION OF T CELL RESPONSES

T cells are key effector cells for anti‐cancer immunity. CD8^+^ cytotoxic T lymphocytes (CTLs) are able to directly lyse tumor cells, produce cytokines that create a pro‐inflammatory environment and activate antigen‐presenting cells (APCs) (Hadrup, Donia, & Thor Straten, 2013). CD4^+^ T cells may be polarized to different subtypes, such as T_H_1, T_H_2 or Tregs (Cook et al., 2012; Swain, 1995). T_H_1 cells produce pro‐inflammatory cytokines that assist CD8^+^ CTLs in killing tumor cells and help creating an environment hostile to the tumor (Knutson & Disis, 2005). Other T_H_ cell subsets, in particular Tregs, are not desired, as they inhibit the activity of tumor killing CTLs (Facciabene, Motz, & Coukos, 2012; Y. Wang et al., 2012). Hence, induction of T_H_1 cells and CTLs is the goal of cancer vaccines.

**Figure 2:**
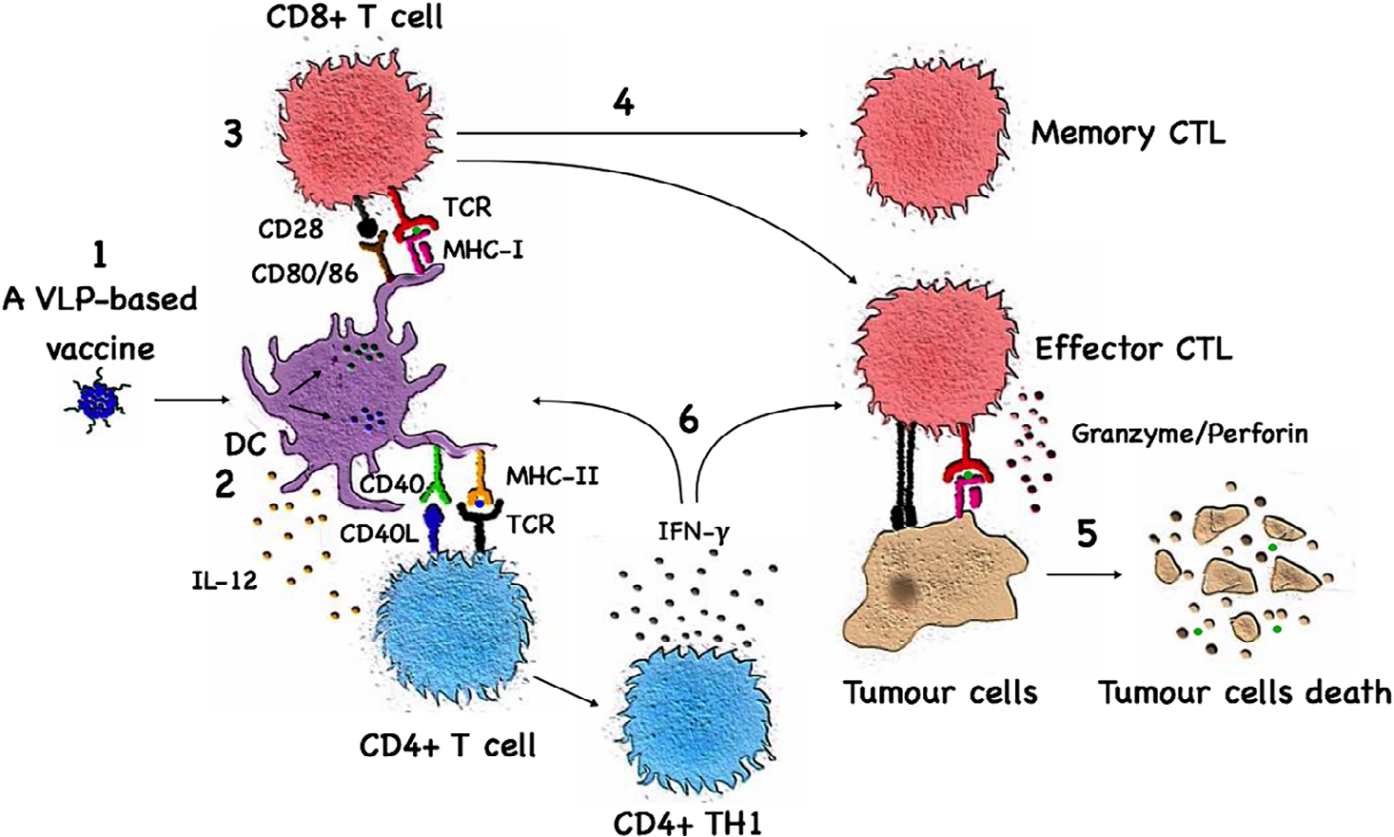
Virus‐like particle (VLP)‐based vaccines and the induction of T cell responses. Following injection, a VLP‐based vaccine is taken up by dendritic cells and macrophages (1). The phagocytosed VLPs displaying the tumor antigen will be processed and presented on both MHC‐II (2) and MHC‐I (3) for recognition by CD4^+^ and CD8^+^ T cells, respectively. Naïve CD8^+^ T cells will proliferate and differentiate into various types of effector and memory cytotoxic T‐lymphocyte (CTLs) (4). CTLs will initiate the killing process of tumor cells (5). Effector CD4^+^ T_H_1 cells enhance antigen presentation by antigen‐presenting cells and assist activated CTLs (6)

VLPs displaying T cell epitopes are capable of eliciting efficient T_H_1 and CTL responses even though they do not carry any genetic material (Deml, Schirmbeck, Reimann, Wolf, & Wagner, 1997; Kazaks, Balmaks, Voronkova, Ose, & Pumpens, 2008; Rueda et al., 1999). While exogenous antigens usually preferably enter the major histocompatibility Class II (MHC‐II) pathway and prime CD4^+^ T cells, VLPs have also the ability to be cross‐presented and enter the major histocompatibility Class I (MHC‐I) pathway (Bell, Young, & Banchereau, 1999; Moron, Rueda, Sedlik, & Leclerc, 2003). This ability is owed to their particulate nature which allows the VLPs to be efficiently taken up by APCs (Figure 2) and enter the MHC‐I pathway via a transporter‐associated with antigen processing (TAP)‐independent endosomal pathway, where MHC‐I molecules are loaded within the endosome as well as a TAP‐dependent endosome to cytosol pathway (Bachmann et al., 1995; Ruedl et al., 2005; Storni & Bachmann, 2004). VLPs combined with stimuli for APCs are able to induce CTL and T_H_1 responses. We have, for example, shown that bacteriophage‐derived Qβ‐VLPs are able to induce CTL and T_H_1 responses when loaded with toll‐like receptor (TLR)‐ligands such as RNA or CpGs, whereas they fail to do so when empty or loaded with polyglutamate (Keller et al., 2010; Schwarz et al., 2005). Packaging CpGs inside VLPs' shell was found to have several advantages: (a) it ensures specific delivery of the CpGs to APCs actually presenting the peptide, (b) it enhances the stability of CpGs, (c) it reduces unfavorable side effects of administering free CpGs and (d) it augments T cell responses (Storni et al., 2004). Interestingly, TLR‐ligands and antigens can be delivered in separate VLPs without the need for physical linkage and still generate a significant CTL response (Gomes et al., 2017; Mohsen et al., 2017). Various adjuvants, in particular those that form a depot or drive additional activation pathways, provide further opportunities to enhance protective T cell responses.

VLPs may be taken up locally at the injection site or freely drain to LNs where they are taken by LN resident APCs (Bachmann & Jennings, 2010; Cubas et al., 2009). Interestingly, both LN resident CD8^+^ and CD8^−^ dendritic cells (DCs) are able to present VLP‐derived epitopes in association with MHC‐II molecules, while CD8^+^ DCs are efficient at cross‐presenting peptides on MHC‐I molecules (Keller et al., 2010). Other types of DCs, such as Langerhans and dermal DCs as well as macrophages have also been reported to cross‐present VLP‐derived antigens (Ruedl et al., 2005; Ruedl, Storni, Lechner, Bachi, & Bachmann, 2002).

We have seen a strong ability of VLPs to boost T cell responses in a homologous fashion both for Qbeta and human papilloma virus (HPV)‐E7 oligomers (Gomes, Flace, et al., 2017; Schwarz et al., 2005). However, we have also seen that passively transferred antibodies (Abs) before priming with VLPs strongly reduces subsequent cross‐priming using the Qbeta platform (Keller et al., 2010). Hence, if T cell responses upon priming are low, Abs may prevent further boosting of the T cell response. With this respect it is interesting to note that HPV VLPs are not loaded with RNA, which may limit T cell immunogenicity upon priming (Da Silva et al., 2003).

## 3 VLP‐BASED VACCINES AND THE INDUCTION OF B CELL RESPONSES

**Figure 3:**
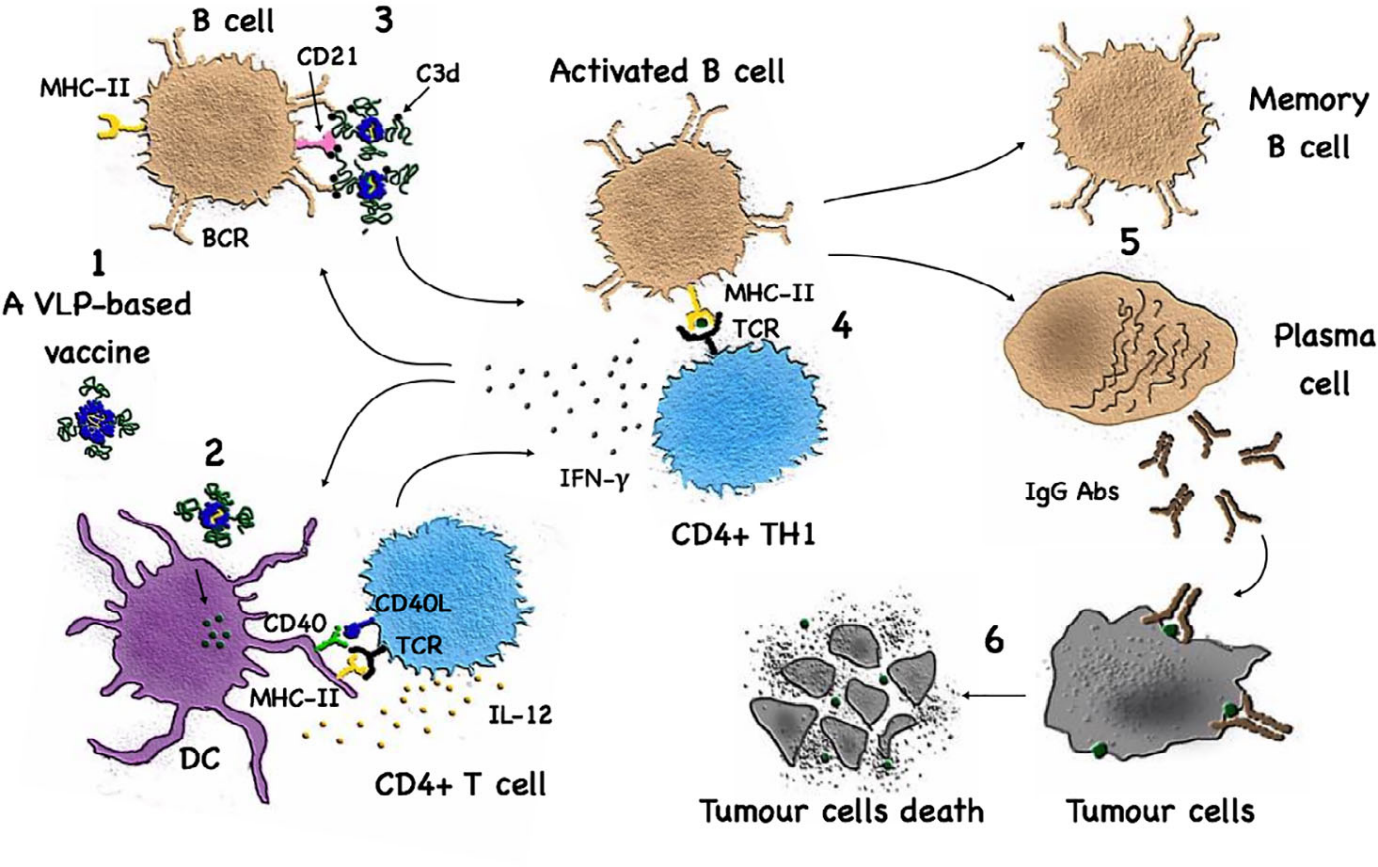
Virus‐like particle (VLP)‐based vaccines and the induction of B cell responses. The highly organized and repetitive surface geometry of a VLP‐based vaccine (1) facilitates its opsonization and phagocytosis by antigen‐presenting cells (2) as well as the engagement of CD21 on B cells (3). B cells also interact with and receive help from T_H_ cells subsequent to uptake of VLP‐based vaccines via B cell receptor (4). This interaction between T_H_ CD4^+^ T cells and B cells are essential for efficient generation of Ab‐producing plasma cells as well as for memory B cells (5). Many VLPs carry RNA packaged during production engaging TLR7/8 in B cells, promoting isotype‐switching towards protective T_H_1 IgG subclasses which will cause tumor cell destruction (6)

Antibodies may also play important roles in anti‐tumor immune responses and have been utilized for the development of effective cancer treatments (Figure 3). The effector killing of Abs can be achieved through different mechanisms such as the induction of apoptosis, abrogation of tumor‐cell signaling pathways, activating complement‐dependent cytotoxicity, regulating T cell function or by antibody‐dependent cellular cytotoxicity (ADCC; Attarwala, 2010; Snijdewint et al., 2001; W. Wang, Erbe, Hank, Morris, & Sondel, 2015; Weiner, Surana, & Wang, 2010). Antigens bound to Abs are taken up by antigen presenting cells via Fc receptors. This enhances MHC presentation and thereby modulates T cell responses. In addition, such immune‐complexes fix complement which causes activation of the APCs, further boosting T cell responses (Platzer, Stout, & Fiebiger, 2014).

Antibodies are the crucial effector molecules induced by the majority of VLP‐based vaccines. The surface of most viruses and bacteria in nature consists of highly organized and repetitive proteins (Bachmann & Zinkernagel, 1997; Feldmann & Easten, 1971). The innate immune system has evolved to recognize such repetitive surfaces and such structures may be viewed as PASP (Bachmann & Jennings, 2011). Most notable, such repetitive patterns can effectively cross‐link B cell receptors (BCRs) and deliver strong activation signals to B cells (Bachmann et al., 1993; Chackerian, Lowy, & Schiller, 2001). This concept also holds true for peptides and proteins linked to VLPs, as up to 300 or 400 peptides can be displayed on a VLP's surface with 5–10 nm distance and thus are capable of eliciting optimal Ab responses (Jegerlehner et al., 2002).

Most antigens targeted by Abs are cell surface molecules. However, cell surface molecules expressed by the host generally induce B cell unresponsiveness (Goodnow, 1996) and conventional vaccination strategies are not able to overcome tolerance and induce Abs specific for cell surface molecules. It is therefore of key importance in this context that highly repetitive antigens as found on the viral surface (Bachmann et al., 1993) or displayed on VLPs (Chackerian et al., 2001; Spohn et al., 2007) can overcome B cell unresponsiveness and induce specific Ab responses against membrane proteins. Indeed, the membrane protein HER‐2 displayed on VLPs was also able to induce specific Abs (Palladini et al., 2018). In addition to Abs‐specific membrane proteins, intracellular proteins may also be an attractive target. Intracellular proteins are an important class of targets as many of the classical cancer testis antigens, for example, MAGE (van der Bruggen et al., 1991) or NY‐ESO‐1 (Jager et al., 1998) is in fact intracellular. Specific Abs may be able to enhance inflammatory processes in the tumor and may also enhance CD4^+^ and CD8^+^ T cell responses by modulating antigen presentation. Again, the unique immunogenicity of antigens displayed on VLPs may be a key for the success of such vaccines.

VLPs displaying self‐antigens have been used preclinically and clinically for successful induction of self‐antigen‐specific Abs that could break self‐tolerance and unresponsiveness in different chronic diseases (Bachmann & Jennings, 2011). Generally, VLPs are efficient in activating complement via the alternative and classical pathways (Barrington, Zhang, Fischer, & Carroll, 2001) facilitating subsequent activation of B cells via the engagement of the CD21/CD19 complexes (Carter & Myers, 2008). The engagement of these receptors enhances the expression of different transcription factors required for the differentiation of long‐lived plasma cells such as X‐box binding protein 1 and B lymphocyte‐induced maturation protein 1 (Gatto et al., 2005; Martins & Calame, 2008). In addition, via binding of natural IgM Abs and recruitment of C1q, B cells can bind VLPs through CD21 and deliver them into B cell follicles for deposition on follicular dendritic cells driving the germinal center reaction (Link et al., 2012). Complement‐decorated viral particles are effectively targeted to secondary lymphoid organs (Ochsenbein et al., 1999) and also stimulate T cell responses (Kopf, Abel, Gallimore, Carroll, & Bachmann, 2002). Many VLPs package RNA during production, which serves as a ligand for TLR7/8 in B cells. This drives isotype switching to more protective IgG subclasses (IgG1 in humans, IgG2a in mice) (Gomes et al., 2019; Bessa & Bachmann, 2010; Jegerlehner et al., 2007), resulting in enhanced ADCC.

## 4 VACCINES IN THE CONTEXT OF CHECK‐POINT INHIBITORS

**Figure 4:**
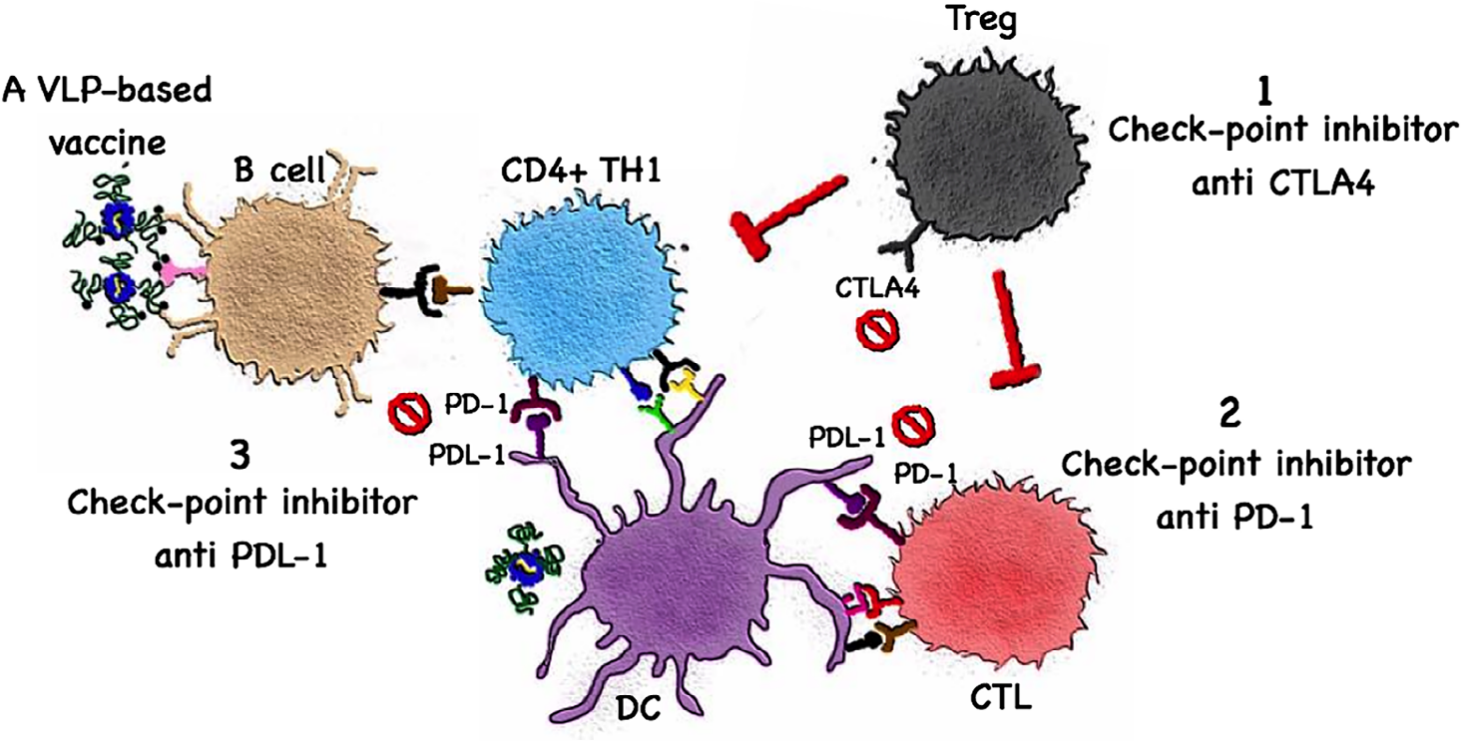
Vaccines in the context of check‐point inhibitors. Cancer immunotherapy targets immune checkpoints that regulates and inhibits the immune system. Monoclonal antibodies targeting (1) CTLA4 molecules on Tregs, for example, Ipilimumab (2) PD‐1 on T cells, for example, Nivolumab or Pembrolizumb (3) PDL‐1 on myeloid cells or/and tumor cells, for example, Atezolizumab have been approved by The food and drug administration (FDA) and are currently used in the clinics for treating different solid tumors. Combining a VLP‐based vaccine with checkpoint inhibitor is an optimal strategy to augment the immune response

Check‐point inhibitors are monoclonal Abs blocking molecules involved in inhibition of T cell activation, enhancing T cell responses and anti‐tumor protection (Figure 4). T cell activation may be inhibited through immune regulation, usually mediated by regulatory T cells (Tregs) (Tanaka & Sakaguchi, 2017). Alternatively, T cells may be blocked to become fully activated through inhibitory receptors or cytokines (Ottaviano, De Placido, & Ascierto, 2019). Ipilimumab serves as a good example for blocking Tregs by targeting the cytotoxic T‐lymphocyte‐associated antigen 4 (CTLA4) while Nivolumab blocks programmed cell death protein 1 (PD‐1), a receptor on T cells that directly blocks T cell activation (Seidel, Otsuka, & Kabashima, 2018). Inhibiting both pathways has so far shown best results in the clinic (Topalian, Taube, Anders, & Pardoll, 2016). Check‐point inhibitors can, however, only amplify preexisting T cell responses. It is therefore rather obvious that combining the vaccination approach with check‐point inhibitors may be an optimal strategy, as the vaccine induces T cell responses which are amplified in their immune attack capacity by the check‐point inhibitors. Indeed, we have recently shown in mice that VLP‐based vaccination against melanoma is much more effective, if Tregs are depleted during therapy (Mohsen, Vogel, et al., 2019).

## 5 VIRUS‐LIKE PARTICLES AS THERAPEUTIC CANCER VACCINES: PRECLINICAL AND CLINICAL STUDIES

Published preclinical and clinical studies have provided notable and convincing evidences that VLPs constitute an immunogenic template for the development of effective therapeutic cancer vaccines. The protective principle induced by VLPs may be based on T cells and/or Ab responses. In this section, we discuss some promising preclinical and clinical studies as summarized in Table 2 in different types of cancers, mainly solid tumors. The use of VLPs as a delivery system for therapeutic drugs is not discussed in this review.

**Table 2:** List of preclinical studies and clinical trials using virus‐like particles (VLPs) as a vaccine template in different types of solid tumors

VLP	Cancer type	Cancer antigen targeted	Adjuvant or combination therapy	Study phase	References
Preclinical studies					
Polyomavirus	Melanoma	OVA (model antigen), TRP2	With or without QuilA‐saponin adjuvant	Preclinical	Brinkman et al. (2005)
Bacteriophage Qβ	Melanoma	PMEL17, MTC‐1, Calpastatin, ZFP518, TRP‐2, Caveolin2, Cpsf3l and Kifl8b	Anti‐CD25	Preclinical	Mohsen, Vogel, et al. (2019)
Cucumber mosaic VLPs	Melanoma	LCMV‐gp33	Microcrystalline tyrosine	Preclinical	Mohsen, Heath, et al. (2019)
Empty cowpea mosaic VLPs	Metastatic models	—	—	Preclinical	Lizotte et al. (2016)
MS2	Breast cancer	xCT	—	Preclinical	Bolli et al. (2018)
AP205	Breast cancer	HER‐2	—	Preclinical	Palladini et al. (2018)
HBcAg	Hepatocellular carcinoma	MAGE‐1, MAGE‐3, AFP‐1	—	Preclinical	H. G. Zhang et al. (2007)
HBcAg	Hepatocellular carcinoma	HBx	—	Preclinical	Ding et al. (2009)
SIV	Pancreatic cancer	Trop2	Alone or with gemcitabine	Preclinical	Cubas, Zhang, Li, Chen, and Yao (2011)
SHIV	Pancreatic cancer	hMSLN	—	Preclinical	Li et al. (2008)
SHIV	Pancreatic cancer	mMSLN	—	Preclinical	S. Zhang et al. (2013)
IBDV	Cervical cancer	E7	—	Preclinical	Martin Caballero et al. (2012)
RHDV	Cervical cancer	E6	Anti‐CTLA4 or anti‐CD25	Preclinical	Jemon et al. (2013)
Bacteriophage Qβ, HBcAg	Fibrosarcoma	LCMV‐gp33	CpG type B	Preclinical	Storni et al. (2004)
Bacteriophage Qβ	Adenocarcinoma	TACAs (Tn)	—	Preclinical	Sungsuwan, Wu, and Huang (2017)
RHDV	Colorectal cancer	Topoisomerase IIα and surviving	Unmethylated CpGs	Preclinical	Donaldson et al. (2017)
Clinical trials					
Bacteriophage Qβ	Melanoma	—	Pembrolizumab (anti‐PD‐1)	Phase I	NCT02680184 (https://www.clinicaltrials.gov/ct2/show/NCT03596476?rank=1)
Bacteriophage Qβ	Malignant melanoma	—	Pembrolizumab (anti‐PD‐1)	Phase I	NCT03084640 (https://www.clinicaltrials.gov/ct2/show/NCT03084640?cond=CMP-001&rank=4)
Bacteriophage Qβ	Melanoma, lymph node cancer	—	Nivolumab (anti‐PD‐1)	Phase II	NCT03618641 (https://www.clinicaltrials.gov/ct2/show/NCT03618641?cond=cmp-001&rank=1)
Bacteriophage Qβ	Melanoma Stage II/IV	Melan‐A	CpG type A	Phase I/II	Speiser et al. (2010)
Bacteriophage Qβ	Melanoma Stage II/IV	Melan‐A	IFA (Montanide), topical Imiquimod +/− IFA	Phase IIa	Goldinger et al. (2012)
Chimaeric HPV16‐VLPs	Cervical intraepithelial neoplasia (CIN 2/3)	E7 and 16L1	—	Phase I	Kaufmann et al. (2007)

### 5.1 VLP‐based vaccines against melanoma

**Figure 5:**
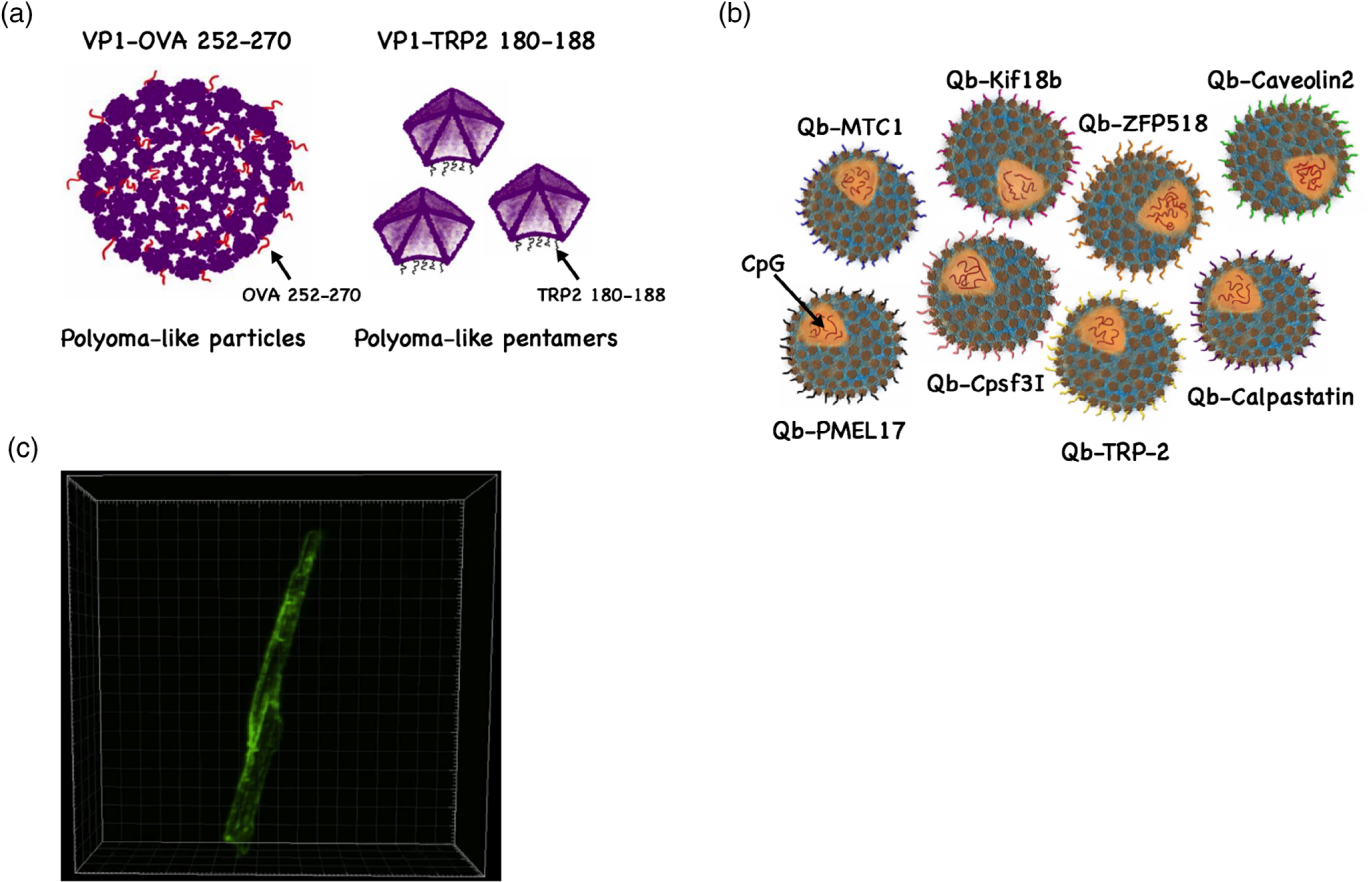
Virus‐like particle (VLP)‐based vaccines against melanoma. (a) (1) Ovalbumin (OVA_257–264_) nonself epitope was fused to the C terminus of VP1 major coat protein of polyomavirus forming polyoma‐like particles of ~45 nm. (2) Tyrosinase‐related protein 2 (TRP2_180–188_) self‐epitope was fused to VP1 major coat protein forming polyoma‐like pentamers of ~9 nm. (b) Mix multi‐target VLP‐based vaccine consisting of germline and mutated CTL epitopes coupled to Qβ‐VLPs by Cu‐free click chemistry and loaded with B‐type CpGs (Mohsen, Vogel, et al., 2019). (c) A microcrystalline tyrosine crystal decorated with CuMV_TT_‐p33 nanoparticles labeled with AF488 (Mohsen, Heath, et al., 2019)

Brinkman et al. (2005) have used the empty polyomavirus‐like particles as a vaccine platform in a melanoma murine model. The study has evaluated the therapeutic value of the particulate structure of the major coat protein VP1 by fusing a nonself or a self‐antigen at the C terminus of VP1. Ovalbumin (OVA_257–264_) was used as a model of nonself‐antigen while tyrosinase‐related protein (TRP2_180–188_) was used as a self‐antigen. Both antigens are H2‐K^b^ restricted T cell epitopes. VP1‐OVA_252–270_ successfully formed VLPs of about 45 nm displaying the epitope inside the particle (Figure 5a‐1), while VP1‐TRP2_180–188_ failed to form full capsid VLPs and resulted in the formation of polyomavirus‐like‐pentamers of about 9 nm (Figure 5a‐2). The immunogenicity of VP1‐OVA_252–270_ VLPs was evaluated by using H2‐K^b^‐OVA tetramers in the splenocytes of immunized mice. The efficacy of the VLP‐based vaccine was measured by assessing survival. VLPs and polyomavirus‐like‐pentamers of VP1‐OVA_252–270_ increased survival by 80–100%. In contrast, the therapeutic treatment with polyomavirus‐like‐pentamers VP1‐TRP2_180–188_ enhanced survival by 40%. The study indicated that both VLPs and polyomavirus‐like‐pentamers are capable of inducing CTL responses in mice (Brinkman et al., 2005). Whether increased efficacy of the OVA‐based vaccine (corresponding to a mutated epitope) compared to the TRP2‐based vaccine (corresponding to a self‐epitope) is due to increased efficacy of the “mutated” epitope or because it assembled into a full VLP remains to be seen.

In 2010, we developed a therapeutic human VLP‐based vaccine Qβ(G10)‐Melan‐A that was tested in Phase I/II study in Stage II/IV melanoma patients. The VLP‐based vaccine was designed by linking the melanoma differentiation specific antigen Melan‐A/Mart1 (The HLA‐A2 restricted epitope extended by a few amino acids) to Qβ‐VLPs which were additionally packaged with the TLR‐9 ligand G10 (a type‐A CpG) as an adjuvant for effective stimulation of DCs and CD8^+^ T cells (Speiser et al., 2010). Type A CpGs were used as they are the strongest inducers of type I interferons which is predominantly produced by plasmacytoid DCs (Pashine, Valiante, & Ulmer, 2005). The results of the study were promising as 63% of the patients generated specific T cell responses capable of producing high cytokine levels (IFN‐γ, TNF‐α, IL‐2), and the responses included central memory T cells. The route of administration (subcutaneous vs. intradermal) did not reveal important differences (Speiser et al., 2010). In addition to strong CD8^+^ T cell responses, Melan‐A‐specific CD4^+^ T cell responses were also induced upon vaccination (Braun et al., 2012). Subsequently, we have conducted a Phase IIa study for Stage II/IV melanoma patients using the same vaccine Qβ(G10)‐Melan‐A, formulated in different adjuvants such as Montanide (incomplete Freund's adjuvant; IFA) or topical Imiquimod and comparing different routes of administration (direct injection into the lymph node [LN], subcutaneous or intradermal). The results showed that 76% of the patients generated specific T cell responses. Using topical Imiquimod as an adjuvant with Qβ(G10)‐Melan‐A vaccine injected subcutaneous or intradermal route was the most effective at inducing central memory T cells. This study concluded that combining CpGs with topical Imiquimod could enhance CD8^+^ central memory T cell responses in melanoma patients (Goldinger et al., 2012). Furthermore, and most remarkably, the study demonstrated by positron emission tomography a long‐lasting immune response for more than a year in draining LNs of 87% of vaccinated individuals. This reveals a sustained immune response induced by the VLP‐based vaccine. Some patients exhibited late onset loss of Melan‐A expression by tumor cells in situ, suggesting that the strong T cell responses provoked outgrowth of antigen loss tumor cell variants, indicating that using a single peptide may not be sufficient to eliminate tumors (Goldinger et al., 2012).

CMP‐001 is the same VLP as described above and therefore is also based on the bacteriophage Qβ‐VLPs loaded with A‐type CpGs. This VLP was formerly called QβG10 and has been successfully used to treat allergies (Klimek et al., 2011) and allergic asthma (Beeh et al., 2013). The same VLP, now called CMP‐001, is being examined for the treatment of cancer. In contrast to conventional vaccines, it does not contain any tumor antigen(s). Three clinical trials are currently ongoing, testing the administration of CMP‐001 as monotherapy or in combination with anti‐PD‐1 check‐point inhibitors (Pembrolizumab or Nivolumab) in melanoma patients; NCT02680184, NCT03084640 and NCT03618641. The recently collected data from the ongoing clinical trial Ib (NCT02680184) showed a manageable toxicity profile and could reverse resistance to check‐point inhibition with significantly increased clinical benefits. The study has been extended and is expected to end in November 2019 (American Association for Cancer Research Annual Meeting, 2018). CMP‐001 is also examined in further trials including patients with NSCLC (NCT03438318), colorectal cancer (CRC) and lymphoma.

Following a similar avenue, Lizotte et al., used an empty Cowpea Mosaic VLPs (eCPMV) in a preclinical model of metastatic cancer. eCPMV is a nonenveloped plant virus that is about 30 nm in size and does not carry any nucleic acids. The study determined whether inhalation of eCPMV could induce anti‐tumor immunity in a lung metastatic B16F10 melanoma murine model. The results revealed that inhalation of eCPMV significantly reduced the tumor burden which was confirmed by assessing tyrosinase expression and metastatic‐like tumor foci in the lungs of B16F10 tumor bearing mice. Furthermore, the inhalation of eCPMV triggered the activation and accumulation of neutrophils in the lungs accompanied with an increase in neutrophil‐associated chemokines and cytokines. The study concluded that the anti‐tumor effects induced by eCPMV required neutrophils. eCPMV was also tested in the 4T1 metastatic lung murine model as well as in disseminated peritoneal ovarian cancer murine model. The results showed again that eCPMV could hinder tumor progression and enhance the survival rate. Furthermore, the authors have shown that intra‐tumoral injection of eCPMV is also effective in treating dermal B16F10 melanomas, forming central tumor necrosis (Lizotte et al., 2016).

We have recently utilized Qβ‐VLPs as a platform to develop a personalized VLP‐based vaccine. Germline and mutated CTL epitopes of B16F10 murine melanoma cell line were identified and predicted by immunopeptidomics and whole exome sequencing, respectively. Corresponding peptides were coupled to Qβ‐VLPs loaded with TLR9 ligands using the bio‐orthogonal Cu‐free click chemistry method, a key step in our novel platform. Three sets of multi‐target vaccines have been prepared, one set was based on identified germline CTL epitopes (GL‐MTV[multi target vaccine]), the second set was based on mutated CTL epitopes (Mutated‐MTV) and the last set combined both types of epitopes (Mix‐MTV) (Figure 5b). Both GL‐MTV and Mutated‐MTV induced protection; however, the best therapeutic effect was achieved by combining both germline and mutated epitopes. The Mix‐MTV could significantly hinder the progression of the aggressive transplanted B16F10 murine melanoma model by enhancement of CD8^+^ T cell infiltration into the tumor. Furthermore, Mix‐MTV affected the myeloid composition of the tumor by increasing the infiltration of Ly6G^+^ granulocytic cells and decreasing the monocytic cells characterized by Ly6C^+^ expression (Mohsen, Vogel, et al., 2019).

In another recent study, we have harnessed the physiological properties of the lymphatic system for better CTL responses when vaccinating with VLPs. Basically, we have formulated cucumber mosaic virus‐derived nanoparticles (CuMV_TT_‐VLPs) incorporating a universal tetanus toxoid epitope TT830–843 and displaying the LCMV‐gp33 peptide with the micron‐sized microcrystalline adjuvant (MCT). Our results show that CuMV_TT_‐p33 nanoparticles decorate the surface of the micron‐sized MCT adjuvant and form a local depot (Figure 5c). Using the stringent B16F10 murine tumor model our results showed that vaccination with this combination enhanced the specific CTL response, mediating strong anti‐tumor effects (Mohsen, Heath, et al., 2019).

### 5.2 VLP‐based vaccines against breast cancer

**Figure 6:**
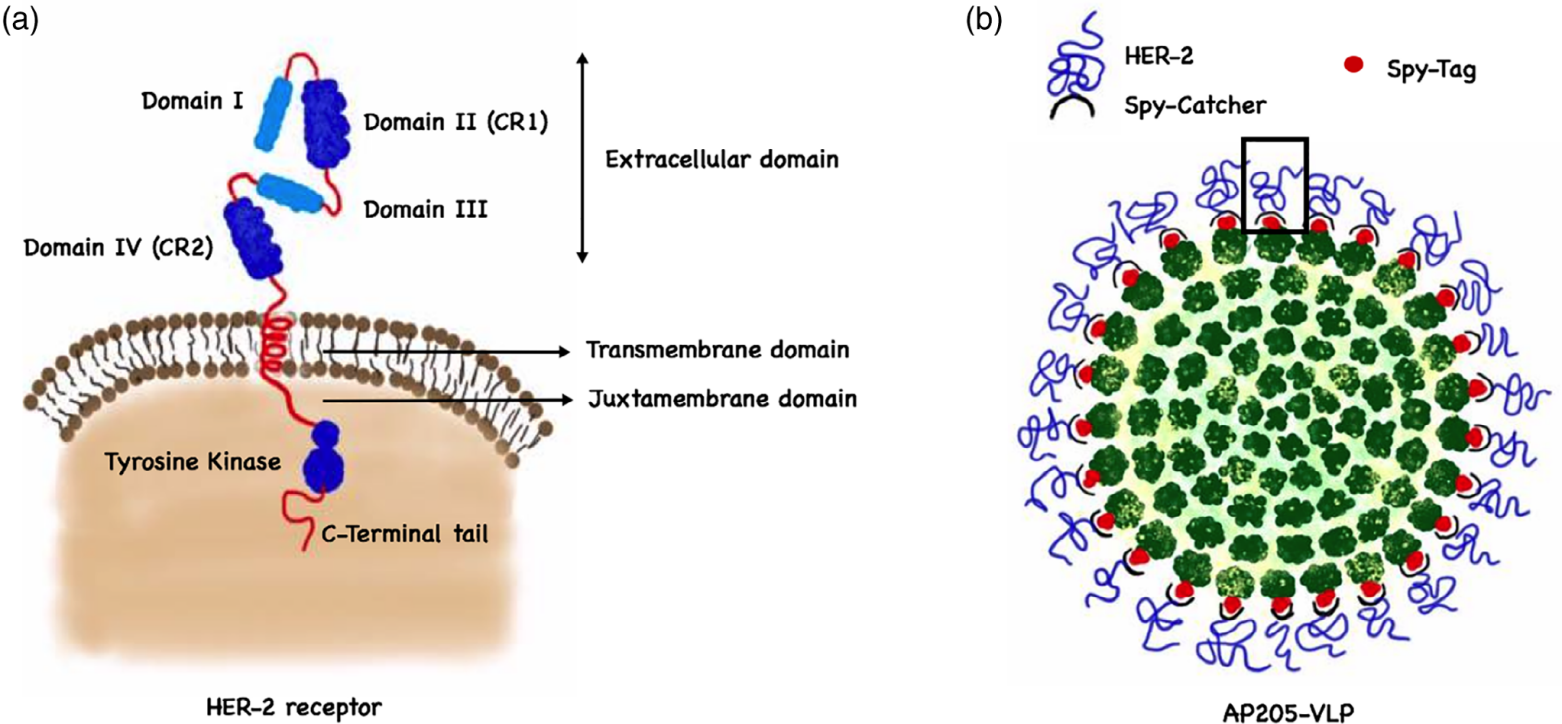
Virus‐like particle (VLP)‐based vaccines against breast cancer. (a) A schematic diagram of the structure of the HER‐2 receptor. The extracellular part consists of four domains: Domain I, Domain II (CR1), Domain III and Domain IV (CR2). The other domains are single transmembrane domain, juxtra membrane domain, tyrosine‐kinase domain and finally a C‐terminal tail. (b) SpyTag/SpyCatcher VLP‐based vaccine based on displaying the subdomains I–IV of HER‐2 combined to SyCatcher covalently attached to AP205‐VLP outer surface fused to a SpyTag part

VLP‐based vaccines have also been tested in breast cancer in several preclinical studies. The human epidermal growth factor receptor 2 “HER‐2 or HER‐2/neu” (Figure 6a) has been shown to be over‐expressed in certain types of aggressive breast cancers and thus constitutes a promising vaccine target (Mitri, Constantine, & O'Regan, 2012).

A recent preclinical study conducted by Bolli et al. (2018) has shown that AX09‐0M6 VLPs derived from the bacteriophage MS2 can efficiently be used as a cancer vaccine platform in some aggressive forms of breast cancers, namely triple‐negative and HER‐2^+^ breast cancer. The study was designed to target the cysteine–glutamate exchanging transporter (xCT), a member of heterodimeric sodium‐independent transporter system that is highly specific for cysteine and glutamate (Lewerenz et al., 2013). xCT protein has been found to be over‐expressed in breast cancer stem cells (Saya, 2014). The AX09‐OM6 vaccine was designed by inserting the six extracellular domain of human xCT protein into MS2‐VLP single coat protein in the AB loop domain. The vaccine elicited a potent IgG2a Ab response directed against the inserted xCT epitope. It was also tested in mice with established 4T1 subcutaneous breast cancer tumors. The results revealed that the vaccine significantly hampered the growth of the established tumors and prevented pulmonary metastases (Bolli et al., 2018).

Palladini et al. (2018) have designed a unique VLP‐based vaccine allowing a directional and high‐density display of the full extracellular domain of HER‐2 (subdomain I–IV) on the surface of AP205‐VLPs. The developed vaccine is based on a SpyTag/SpyCatcher conjugation system where HER‐2 antigen was genetically fused to the SpyCatcher sequence which is attached to the surface of AP205‐VLPs expressing the “SpyTag” (Figure 6b). The study demonstrated that the vaccine platform could overcome B cell tolerance and induced potent anti‐HER‐2 IgG titers. The therapeutic effect of the vaccine was tested in wild type mice grafted with HER‐2 mammary carcinoma cells. The induced Ab titers could effectively hinder tumor progression. The vaccine was also successfully tested as a prophylactic vaccine in transgenic mice spontaneously developing mammary carcinoma expressing HER‐2 (Delta 16 isoform).

### 5.3 VLP‐based vaccines against pancreatic cancer

Development of therapeutic cancer vaccines is considered promising for the treatment of pancreatic cancer (Laheru & Jaffee, 2005). Some studies have tested peptide vaccines, or whole‐tumor vaccines targeting some key signaling proteins such as K‐RAS, vascular endothelial growth factor or epidermal growth factor receptor (Jaffee et al., 2001). Li et al. (2008) have developed a chimeric VLP‐based vaccine by incorporating human mesothelin into simian‐human immunodeficiency virus VLPs. The target epitope was chosen based on previous studies indicating a role of MSLN in tumor adhesion and dissemination (Rump et al., 2004). The results showed that vaccination with the chimeric VLP‐based vaccine substantially inhibited progression of an orthotopic pancreatic tumor in mice. The developed vaccine increased CTL activity and caused reduction in regulatory T cells (Tregs; CD4^+^FOXP3^+^) which correlated with enhanced survival. In a next step, the group has further evaluated whether the incorporation of murine mMSLN into VLPs could break tolerance to self‐mMSLN and allow to mount an effective immune response capable of controlling tumor progression. Their results showed that immunization with mMSLN‐VLP vaccine can break the tolerance towards the self‐MSLN and induce specific CD8^+^ T cell response leading to tumor regression in an orthotopic pancreatic cancer murine model (Li et al., 2008).

In a similar study, mTrop2 was incorporated into the surface of an enveloped simian immunodeficiency virus‐VLP. The mTrop2‐VLP vaccine was tested in a syngeneic murine pancreatic cancer model (Cubas et al., 2011). The results were promising as a significant reduction in tumor size was accompanied by an increase in the infiltration of mTrop2‐specific CD8^+^ T cells, CD4^+^ T cells as well as natural killer cells. Furthermore, immunization with mTrop‐VLP caused a decrease in Tregs and myeloid‐derived suppressor cells in the tumor microenvironment (Cubas et al., 2011). The study also showed that combining the vaccine with gemcitabine—a chemotherapeutic drug—can significantly improve the survival of tumor bearing mice.

### 5.4 VLP‐based vaccines against cervical cancer

HPV oncogenes are attractive targets for the development of a therapeutic vaccine. Kaufmann et al. (2007) showed the results of a clinical trial where patients with high‐grade cervical intraepithelial neoplasia (CIN 2/3) were treated with HPV16 L1E7 CVLP as a prophylactic and a therapeutic vaccine. CVLP is a chimeric VLP vaccine consisting of truncated L1 protein at the C‐terminus linked to the N‐terminal part of the E7 protein (E7_1–55_) of HPV16. The main aim of the study was to assess the vaccine's safety and efficacy in HPV16^+^ patients. The results showed that CVLP has a very good safety profile and could induce high Ab titers against L1 and low Ab titers against E7 as well as CTL responses against both proteins. About 39% of the vaccinated patients showed histological improvement to (CIN 1 or normal) versus 25% in the placebo group. Furthermore, 56% of the responders were HPV16 DNA‐negative by the end of the trial.

**Figure 7:**
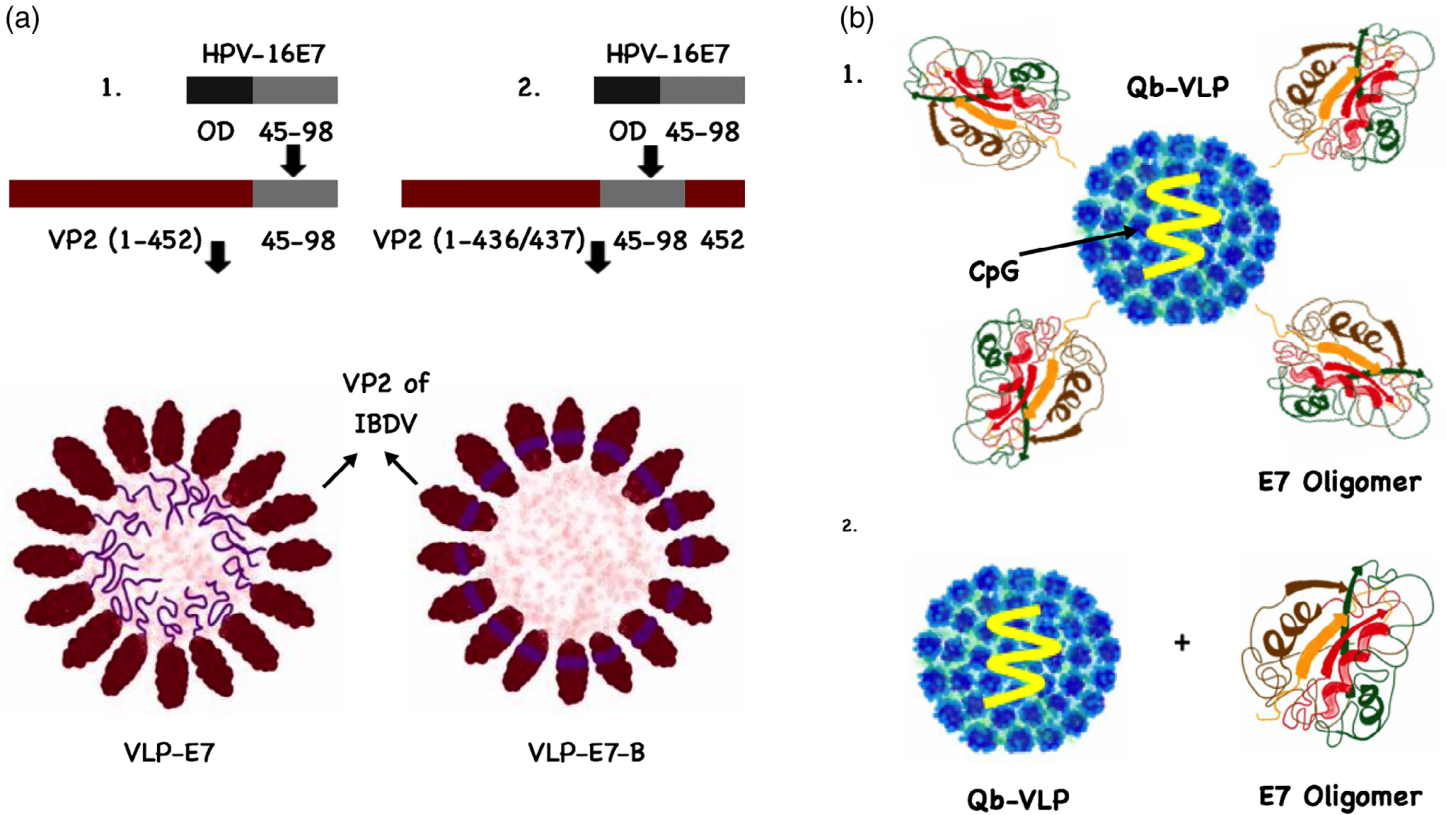
Virus‐like particle (VLP)‐based vaccines against cervical cancer. (a) A sketch illustrates human papilloma virus (HPV) VLP‐based vaccines: (1) VLP‐E7 was based on cloning the C‐terminal region of HPV‐E7 epitope “45–98” excluding the oncogenic domain OD to the C‐terminal of VP2 of IBDV‐VLP, while (2) VLP‐E7‐B was generated by inserting the HPV‐E7 epitope into the VP2 protein. (b) (1) E7 oligomers “50 nm” chemically coupled to Qβ‐VLPs “25–30 nm” using SMPH cross‐linker and loaded with nonmethylated CpGs, (2) E7 oligomers admixed with Qβ‐VLPs loaded with CpGs

Martin Caballero et al. (2012) have developed a preclinical vaccine model by genetically fusing the E7 protein of HPV to VP2 of the infectious bursal disease virus‐VLPs (IBDV) with T1 symmetry (i.e., 60 subunits). The generated VLPs were DNA‐free. Two chimeric VLP‐based vaccines were developed, VLP‐E7 with E7 on the surface and VLP‐E7‐B displaying the heterologous E7 sequence inside the VLP (Figure 7a). The vaccine was tested in HLA‐A2 transgenic mice engrafted with the TC‐1/A2 cervical cancer cell line expressing E7 protein. The results showed that the VLP‐E7 vaccine could elicit IFN‐γ secreting T cells against HPV T cell epitopes even without the use of an adjuvant. VLP‐E7 and VLP‐E7‐B vaccines revealed similar immunogenicity and efficacy indicating that the fusion site is not important. Furthermore, immunizing mice with VLP‐E7, 5 or 12 days post‐tumor cell line inoculation yielded 100% protection. The vaccine was also tested in more stringent situations against preexisting 200–300 mm^3^ tumors and showed complete eradication of the established tumors as well as enhancing the survival of the mice.

Jemon et al. (2013) have used in their study a modified rabbit hemorrhagic disease virus‐VLP (RHDV‐VLP) decorated with the E7_48–57_ peptide. The developed vaccine was tested in combination with anti‐CTLA4 or anti‐CD25 Abs to deplete Tregs in preexisting TC‐1 tumors expressing E6 and E7 in mice. The tumor burden was efficiently reduced by 50% with increased median survival of HPV tumor‐bearing mice.

Gomes, Flace, et al. (2017) have recently demonstrated that the physical linkage of antigens and adjuvants is not necessary for successful T cell activation in a therapeutic vaccine. The study has used E7 protein oligomers with a size of about 50 nm derived from HPV. E7 oligomers were chemically coupled to Qβ‐VLPs loaded with nonmethylated CpGs or simply admixed (Figure 7b). The study has shown that both formulations are capable of inducing protective CD4^+^ and CD8^+^ T cell responses as therapeutic vaccines in an HPV mouse model. The results also revealed that the physical linkage is, in contrast to T cells, necessary for generating B cell responses. The explanation for this unexpected finding was that particles of similar size drain to the same DCs, even if not physically linked.

### 5.5 VLP‐based vaccines against hepatocellular carcinoma

Data from H. G. Zhang et al. (2007) have shown that a chimeric VLPs can be generated using a truncated HBc‐VLP. Four different insertions of common hepatocellular carcinoma (HCC) epitopes; MAGE‐1 (278–286), MAGE‐3 (271–279), AFP1 (158–166) and AFP2 (542–550) were fused to the 3′ end of the truncated HBc‐VLPs. The developed vaccine was tested for its immunogenicity and efficacy in the B16‐pIR‐HH murine tumor model. In this study, DCs were pulsed with the generated chimaeric HBc‐VLP vaccine resulting in stronger CTL responses when compared to DCs pulsed only with peptides. The vaccine could inhibit the growth of the aggressive B16‐pIR‐HH tumor and prolonged the survival of the immunized mice (Y. Zhang et al., 2007).

Ding et al. (2009) developed a multi‐epitope peptide‐loaded VLP vaccine. This was carried out by the insertion of four HBx‐dominant CTL epitopes into the HBV core protein. HBx is the abbreviation for HBV X multifunctional regulatory protein that has been shown to participate in viral oncogenesis. The expression of this gene induces the formation of liver cancer in transgenic mouse models (Cheng, Hu, King, Jay, & Campbell, 1997; Chu, 2000; Kim, Koike, Saito, Miyamura, & Jay, 1991). The inserted HBx epitopes are namely HBx (52–60), (92–100), (115–123) and (140–148). DCs from HLA‐A*0201 transgenic mice or peripheral blood lymphocytes from patients infected with HBV who are HLA‐A2^+^/HBx^+^ were pulsed with HBx_(115–123)_, HBx_(92–100)_, HBx_(140–148)_, or HBx_(52–60)_ and CTL response was measured. The results of the study have shown that the developed multi‐epitope VLP‐based vaccine was significantly more immunogenic and induced stronger anti‐tumor protection than using a single peptide epitope.

### 5.6 More VLP‐based vaccines against cancer

Storni et al. (2004) have shown that peptides chemically coupled to Qβ‐VLPs or genetically fused to the hepatitis B core antigen (HBc‐Ag) and loaded with nonmethylated CpGs can induce powerful CD8^+^ T cell responses in mice. They have used the H2‐D^b^ restricted T cell epitope LCMV‐gp33 as a model antigen. Both types of VLP‐based vaccines hindered the growth of fibrosarcoma tumor cell lines transfected with the relevant epitope in a challenging therapeutic model.

Tumor‐associated carbohydrate antigens (TACAs) are over‐expressed in different types of cancers and are associated with greater progression and metastasis (Monzavi‐Karbassi, Pashov, & Kieber‐Emmons, 2013; Yin & Huang, 2012). TACAs are mainly recognized by BCRs on B cells. Scientists have utilized VLPs as a vaccine template to display TACAs. Specifically, the Tn antigen “N‐acetylgalactosamine monosaccharide structure” has been chemically coupled to Qβ‐VLPs resulting in the display of about 300–400 copies of Tn antigens on the VLPs' surface (Sungsuwan et al., 2017). The results of the study indicated that Qβ‐Tn vaccine can elicit significantly higher IgG Ab titers when compared to the control group immunized with Qβ‐VLPs only without Tn antigen. Furthermore, the induced Abs could recognize tumor cells expressing Tn antigen and protected the mice from the growth of murine mammary adenocarcinoma in a therapeutic setting (Sungsuwan et al., 2017).

The therapeutic potential of VLPs has also been tested in a CRC murine model using chimeric RHDV‐VLPs containing Topoisomerase IIα (topIIα) and/or survivin epitopes derived from CRC tumor‐associated antigens. The multi‐target VLP vaccine appeared more efficacious than the mono‐target vaccine with trends in further enhancing the survival and delaying the tumor growth. These results indicate that targeting more than one epitope may reduce the tumors' capacity to evolve and escape. Interestingly, the mice which achieved complete remission after vaccination were protected from a subsequent tumor rechallenge (Donaldson et al., 2017).

## 6 CHALLENGES FOR DEVELOPING VLP‐BASED CANCER VACCINES

With increasing capacities in recombinant technologies, the future will likely see a large number of VLP‐based cancer vaccines entering clinical development. However, several challenges are threatening the effective development of VLP‐based vaccines. For example, the genetic fusion of epitope insertions into VLPs may result sometimes in misfolding of the particles preventing proper assembly (Sander & Lollini, 2018; Shiyu Dai & Deng, 2018). It may be challenging to achieve an optimal homogenous and high‐density display of the epitopes on the VLPs' surface when using the chemical coupling methods. These problems are accelerated by the fact that T cell epitopes usually are hydrophic and prone to cause aggregation. In addition, the selected coupled or fused epitopes may not induce optimal immune responses for protection against tumors. Finally, high reliable preclinical (murine) models are missing.

## CONFLICT OF INTEREST

M.O.M. and M.F.B. own shares of DeepVax GmbH involved in developing virus‐like particles‐based vaccines for cancer. D.E.S. and A.K. have declared no conflicts of interest.
